# Measles Virus Imported by International Traveler in Jiangsu Province of China, in 2018 and 2019

**DOI:** 10.1155/2020/7318582

**Published:** 2020-02-08

**Authors:** Ying Hu, Xiuying Deng, Peishan Lu, Hongxiong Guo

**Affiliations:** Jiangsu Provincial Center for Disease Control and Prevention, Nanjing, China

## Abstract

Measles remains a public health concern in many regions, and the imported measles cases continue to challenge the measles elimination program for most of the countries where measles was verified to be eliminated or approaching elimination. The imported measles cases have been reported since October, 2017, in Jiangsu province, China. In this study, we reported the first imported B3 genotype measles virus from Egypt and the second imported D8 genotype measles virus from Philippines through international traveling. No secondary measles cases were found after these imported cases. Our findings highlighted the importance of measles vaccination targeting international travelers in China.

## 1. Introduction

Measles is a highly contagious viral disease characterized by fever, cough, coryza, and conjunctivitis followed by characteristic rash [1]. It is still responsible for more than 100 000 deaths every year, although the attenuated measles vaccines are widely used in routine immunization programs and mass vaccination campaigns that were implemented in the world [2]. Even in Latin America where measles has been ever recognized to be eliminated, the outbreaks of measles are common in recent years [3, 4]. The epidemiological evidence showed that international air travelers played an important role on measle outbreak in high vaccine coverage regions. For example, 26% of measles cases were international, imported in USA [5]. Almost all measles viruses circulating in Taiwan province of China were imported nowadays [6]. A new strain of measles, D4-Hamburg, was imported from London to Germany, Hamburg, in December 2008 and subsequently spread and caused an outbreak of >24,300 cases in Bulgaria [7].

Jiangsu province, located in the east of China, is one of economically developed provinces in China. The number of the international travelers has been increasing in past decades. By the end of 2018, it reached 6.7 million travelers. Especially in recent years, more and more people are traveling to Africa and east-south Asia due to the low cost. Before 2017, H1a genotype was the unique virus circulating in Jiangsu province. Until October of 2017, Deng et al. identified two epidemiologically linked cases caused by D8 genotype measles virus, and the index case might be a student from Lao People's Democratic Republic who studied at college in Jiangsu province [8]. From then, two imported measles cases were reported again in Jiangsu province within two years. In this study, we reported molecular epidemiology features of them.

## 2. Methods

### 2.1. Epidemiology Investigation

After the local Center for Disease Control and Prevention received the report of measles case from local hospital by Epidemic Surveillance System, the epidemiology investigation was conducted within 48 hours, which included the collection of demographics, epidemiologic, and clinical data. The Chinese National Notifiable Disease Reporting System was established in 1955. In 1997, China developed a case-based, laboratory-supported measles surveillance system. The two surveillance systems were unified in 2009. Every suspected case is investigated by county-level China CDC staff members using a standardized, in-person questionnaire; outbreaks are investigated and reported by local China CDC staff members. At the same time, the specimens are collected and transported to measles network lab to conduct IgM antibody and nucleic acid test.

### 2.2. Laboratory Test

IgM antibody and nucleic acid of measles virus test were conducted in local county and prefecture center for disease control and prevention according to standard SOP developed by Chinese Center for Disease Control and Prevention, respectively. The genotyping of Mev was conducted in Jiangsu Provincial Center for Disease Control and Prevention. The QIAamp Viral RNA Mini Kit (Qiagen, Hilden, Germany) was used to extract measles virus (MeV) nucleic acid according to the manufacturer's instructions. Reverse transcription amplification was performed using previously described primers to amplify a 634-bp fragment of the N gene [8], which included enough length fragment recommended for genotyping. The PCR products were sequenced using the ABI 3730 DNA Sequencer (Applied Biosystems, Foster City, CA, USA) at Sagon Biotech (Shanghai, China) using directional primers.

### 2.3. The Phylogenetic Analysis

The sequences were manually edited using BioEdit version 7.2.5 software and then analyzed using MEGA 7.0 software. The reference strains include the reference strains recommended by WHO and D8 and B3 genotype sequences isolated from China. The phylogenetic tree was constructed using the neighbor-joining method with 1000-replicate bootstrap test. The two sequences were submitted to GenBank to do Blast to seek the sequences with the highest identity.

## 3. Results

### 3.1. Epidemiology Investigation

**Case 1 .**

, male, 55 years old. He traveled to Egypt during October 31st to November 10th, 2018. He had onset of maculopapular rash on his legs and back on the night of November 8^th^ and then had fever (38°C) on November 10th, which developed into conjunctival congestion on November 12th, and cough on 13th. He was admitted to a hospital on 16th and diagnosed with measles on November 19th. WBC was 6.19 × 109/L (75% neutrophils), aspartate aminotransferase 65.0 U/L, C-reaction protein 23 mg/L, urine occult blood 2+, urine protein +, HBsAg+, HBeAb+, and HBcAb+. Nucleic acid of measles virus is positive using real-time fluorescence PCR test. Among this tour group, none except him was diagnosed as measles. He did not receive any doses of measles-containing vaccine.

**Case 2 .**

, male, 23 years old. He had fever of 38°C in Philippines on March 4^th^, 2019, and rash on March 8^th^; subsequently the rash extended to most of the body skin. He returned to China on the night of March 11th. He was diagnosed as measles in a local hospital on March 12th; the fever disappeared on March 12th. Blood and nasopharyngealswab specimens were collected on March 14^th^ for IgM antibody of measles virus and nucleic acid of measles virus test. Results showed that both of IgM antibody against measles virus and nucleic acid of it were positive. His immunization status was unknown.

### 3.2. The Phylogenetic Analysis

**Figure 1:**
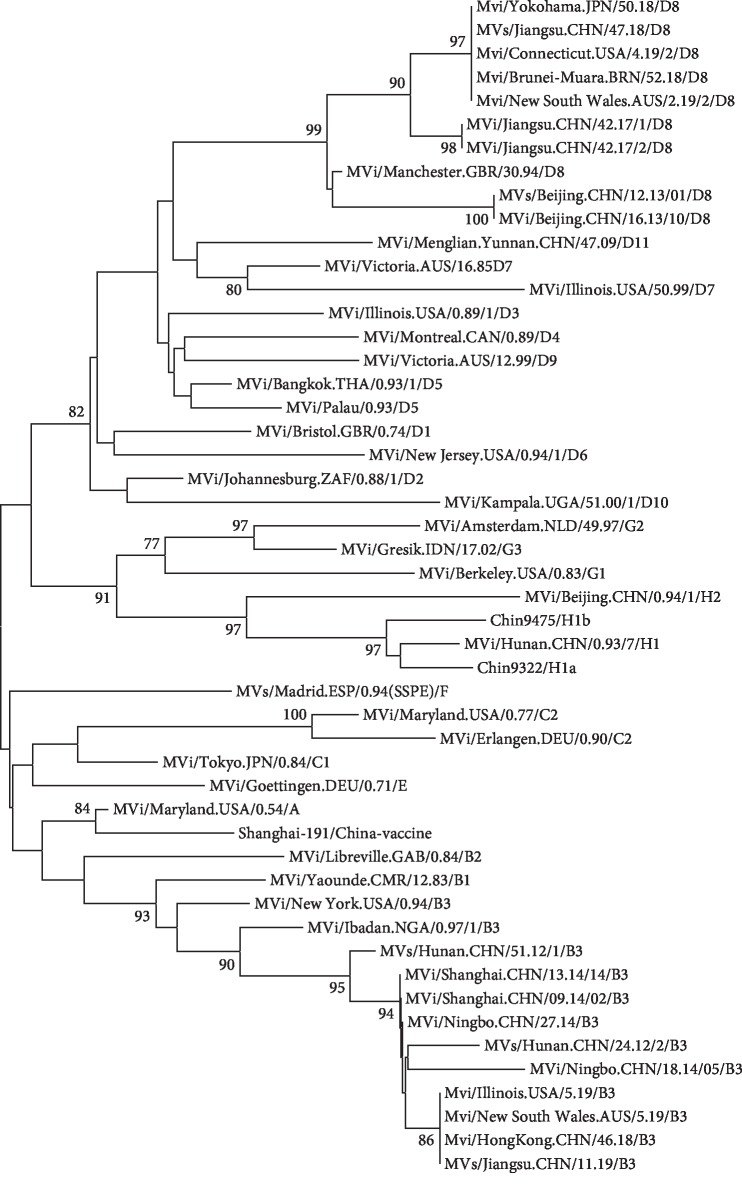
Phylogenetic tree based on the nucleotide protein N gene sequences of various strains of the measles virus using the neighbor-joining method. Numbers at each branch indicate the bootstrap values of the clusters supported by that branch, only more than 75% of bootstrap value is indicated. The sequences isolated from Jiangsu were underlined.

As shown in Figure 1, the sequence of case 1 (MVs/Jiangsu. CHN/47.18/D8) belongs to D8 genotype. It is clustered closely with the sequences isolated from Japan (Yokohama), USA (Connecticut), Brazil (Brunri-Muara), and Australia (New South Wales), not with D8 sequences isolated in 2017 in Jiangsu province. This affirmation is supported by high bootstrap value of 89%. The sequence of case 2 (MVs/Jiangsu. CHN/11.19/B3) belongs to B3 genotype. It is clustered closely with the sequences isolated from USA, Australia, and Hong Kong of China. This affirmation is supported by a high bootstrap value of 100%.

## 4. Discussion

In this study, we reported the first imported B3 genotype measles virus and the second imported D8 genotype measles virus in Jiangsu province. Two patients were infected with measles virus abroad during the international trip, and the time of onset was before they returned to China. After onset of measles, they returned to China and subsequently were diagnosed with measles at local hospitals. Case 1 was infected with D8 genotype measles virus which had higher identity with virus from Japan (Yokohama), USA (Connecticut), Brazil (Brunri-Muara), and Australia (New South Wales) than those isolated from China. Case 2 was infected with B3 genotype measles virus which had higher identity with virus form USA, Australia, and Hong Kong of China. During the investigation of these imported measles cases, no close contacts were diagnosed with the virus. In Jiangsu province, the average measles incidence is about 12 per million people in recent year [9] and vaccination with two doses of measles-containing vaccine covers more than 95% of population [10]. Antibody level in human body often wanes over time. Our previous study also showed that antibody of measles virus was lowest among population aged over 15 years [10]. Nonetheless, there are still some adults and children less than eight months old who did not receive measles vaccine. The gap in measles vaccination may persistently exist. Molecular epidemiology surveillance showed that multiple imported viruses have been detected since 2009 in Beijing, Taiwan, Shandong, and Liaoning of China [11–14]. All the above suggests that the imported viruses via international travelers may cause measles outbreak, even in regions and places, with high measles vaccine coverage. For example, the imported D8 genotypes measles virus caused the measles outbreak in Beijing in 2014. To avoid imported measles, some countries (such as USA) require that someone who travel internationally should receive one dose of MMR vaccine prior to travel. This measure may be also applicable for China with tremendous international travelers due to the increasing measles cases all over the world in recent years [15].

Herein, the evidence obviously supported that case 1 got measles virus in Egypt and case 2 in Philippines. Whether case 1 or case 2, both were infected with measles virus and had onset of measles abroad. Particularly, measles was outbreaking in Philippines when case 2 was staying there. However, it is regretful that no sequences of measles virus of these two countries were found in GenBank database. Phylogenetic analysis cannot provide direct evidence to confirm the source of measles virus in these two cases. The global distribution of measles virus has the regional specificity; the different genotype of measles viruses circulates in various regions [16]. However, the diversity will change and even disappear with frequent international population travel. Although measles virus molecular epidemiology surveillance was recommended by WHO and has been conducted in many countries since the 1990s, the sequences of measles virus that can be gotten are still not enough in public database.

There were several limitations. Firstly, the epidemiology investigation was not conducted among the people taking the same flight when case 1 returned to China. Therefore, it was not clear whether measles virus was transmitted between them. Secondly, in the tour group case 2 joined, a girl even had fever for two days and self-cured. No epidemiology investigation and lab test were conducted for her. So, it is difficult to confirm whether she had measles or not.

Measles virus will continue to be imported into China if measles remains endemic around the world and international travelers increase. Our findings emphasize the importance of maintaining a high level of measles immunity in China populations (measles vaccination of susceptible individuals should include those before international travel) and necessity of widely conducting molecular surveillance in the world and the importance of sharing measles sequence data more widely and timely.

## Data Availability

The data used to support the findings of this study are available from the corresponding author upon request.
